# Safety and efficacy of dual PI3K-δ, γ inhibitor, duvelisib in patients with relapsed or refractory lymphoid neoplasms: A systematic review and meta-analysis of prospective clinical trials

**DOI:** 10.3389/fimmu.2022.1070660

**Published:** 2023-01-04

**Authors:** Zhongwang Wang, Hui Zhou, Jing Xu, Jinjin Wang, Ting Niu

**Affiliations:** Department of Hematology, West China Hospital, Sichuan University, Chengdu, Sichuan, China

**Keywords:** dual PI3K-δ, γ inhibitor, duvelisib, lymphoid neoplasms, safety, efficacy, meta-analysis

## Abstract

**Background:**

Duvelisib is the first FDA-approved oral dual inhibitor of phosphatidylinositol-3-kinase PI3K-delta (PI3K-δ) and PI3K-gamma (PI3K-γ). Although many clinical studies support the efficacy of duvelisib, the safety of duvelisib remains with great attention. This systematic review and meta-analysis aimed to evaluate the safety and efficacy of duvelisib in treating different relapsed or refractory (RR) lymphoid neoplasm types.

**Methods:**

We searched prospective clinical trials from PUBMED, EMBASE, Cochrane Library, and ClinicalTrials.gov. For efficacy analysis, Overall response rate (ORR), complete response rate (CR), partial response rate (PR), rate of stable disease (SDR), rate of progressive disease (PDR), median progression-free survival (mPFS), 12-/24-month PFS, and 12-month overall survival (OS) were assessed. For safety analysis, the incidences of any grade and grade ≥3 adverse events (AEs), serious AEs, and treatment-related discontinuation and death were evaluated. Subgroup analysis based on the disease type was performed.

**Results:**

We included 11 studies and 683 patients, including 305 chronic lymphocytic leukemia/small lymphocytic lymphoma (CLL/SLL), 187 B-cell indolent non-Hodgkin lymphoma (iNHL), 39 B-cell aggressive non-Hodgkin lymphoma (aNHL), and 152 T-cell non-Hodgkin lymphoma (T-NHL) patients. The pooled ORR in CLL/SLL, iNHL, aNHL and T-NHL was 70%, 70%, 28% and 47%, respectively. Additionally, the pooled ORR in CLL/SLL patients with or without TP53 mutation/17p-deletion (62% vs. 74%, p=0.45) and in follicular lymphoma (FL) or other iNHL (69% vs. 57%, p=0.38) had no significant differences. Mantle cell lymphoma (MCL) patients had higher pooled ORR than other aNHL (68% vs. 17%, p=0.04). Angioimmunoblastic TCL (AITL) patients had higher pooled ORR than other PTCL patients (67% vs. 42%, p=0.01). The pooled incidence of any grade, grade ≥3, serious AEs, treatment-related discontinuation and death was 99%, 79%, 63%, 33% and 3%, respectively. The most frequent any-grade AEs were diarrhea (47%), ALT/AST increase (39%), and neutropenia (38%). The most frequent grade ≥3 AEs were neutropenia (25%), ALT/AST increased (16%), diarrhea (12%), and anemia (12%).

**Conclusion:**

Generally, duvelisib could offer favorable efficacy in patients with RR CLL/SLL, iNHL, MCL, and AITL. Risk and severity in duvelisib treatment may be mitigated through proper identification and management.

## Introduction

Lymphoid neoplasms comprise a heterogeneous group of lymphoproliferative malignancies with a variety of clinical, morphologic, and molecular features, for which about 150,000 new cases and 40,000 deaths expected to occur in 2022 in the United States alone (1). Mature B-cell neoplasms and mature T-cell neoplasms represent the most typical lymphocytic tumors originating from cells at stages of maturation after stem cell differentiation (2). Mature B-cell neoplasms account for nearly 65% of all lymphoid neoplasms, with both aggressive and indolent subtypes (2). The latter mainly including chronic lymphocytic leukemia/small lymphocytic lymphoma (CLL/SLL), Follicular lymphoma (FL), Marginal zone lymphoma (MZL), Lymphoplasmacytic lymphoma (3).Widespread use of chemoimmunotherapy has greatly improved the survival of patients with CLL/SLL and indolent non-Hodgkin lymphomas(iNHLs). However, these diseases are currently incurable (4–9). Patients with aggressive B-cell lymphoma(aNHLs), such as diffuse large B-cell lymphoma (DLBCL) and mantle cell lymphoma (MCL), are often diagnosed with advanced stage, and a substantial proportion of patients are refractory to initial chemotherapy or relapse in early years. High-dose chemotherapy with autologous stem cell transplantation may be the only curative choice for patients with relapsed/refractory (R/R) aNHLs. Radiotherapy and single-agent therapies, which play roles in the treatment of iNHL, have shown low expected response rates and duration of responses (10–16). Mature T-cell non-Hodgkin lymphomas (T-NHLs) are another heterogeneous group representing approximately 6% of all lymphoid neoplasms (2), usually including cutaneous T-cell lymphoma (CTCL) and peripheral T-cell lymphoma (PTCL). With a relatively poor prognosis, PTCL includes PTCL not otherwise specified (NOS), angioimmunoblastic T-cell lymphoma (AITL), anaplastic large-cell lymphoma (ALCL), and others (17). In contrast to mature B-cell neoplasms, T-cell lymphomas, especially PTCL, have a high failure rate of first-line chemotherapy, prone to relapse, and lacking an effective monoclonal antibody, like anti-CD20 in B-cell lymphoma (18, 19). Some agents have been approved by the FDA for relapsed PTCL, unfortunately with low response rates and short median progression-free survival (PFS) (20–22). New and more effective agents with distinct mechanisms are urgently needed for patients with relapsed or refractory mature lymphoid neoplasms, whether the tumor originates from B cells or T cells.

Duvelisib is the first FDA-approved oral dual inhibitor of phosphatidylinositol-3-kinase PI3K-delta (PI3K-δ) and PI3K-gamma (PI3K-γ) and shows great potential in many clinical trials for the treatment of relapsed or refractory lymphoid neoplasms, including CLL/SLL, iNHLs, and T-NHLs (23–33). Phosphatidylinositol 3-kinase (PI3K) is a lipid kinase involved in lots of signal transduction. Class I PI3K consists of four catalytic subunits (α, β, γ, δ) in human cells. PI3K-α and PI3K-β show a broad tissue distribution. In contrast, the PI3K-δ and PI3K-γ isoforms are primarily expressed in leukocytes, extensively regulating both innate and adaptive immune function in lymphocyte and myeloid cell function (34–39).PI3K-δ inhibition directly targets proliferation and survival of lymphoid neoplasm cells, while PI3K-γ inhibition reduces the differentiation and migration of crucial tumor support cells in the tumor microenvironment, such as Treg cells and M2 tumor-associated macrophages (33, 40–44). With dual inhibition of PI3K-δ and PIK3-g in preclinical models of CLL, B-cell lymphomas, and T-cell lymphomas, duvelisib showed more robust anti-tumor activity than inhibitors of PI3K-δ isoform alone (33, 44–46).

However, due to the heterogeneity of lymphoid neoplasms, the efficacy of PI3K inhibition in lymphoid neoplasms ranged widely. Meanwhile, the PI3K/AKT/mTOR regulates a range of cellular activities, whether in malignant or normal cells, so off-target effects and side effects are inevitable (47, 48). Some of the toxic effects reported in clinical trials of PI3K-δ inhibitors, incredibly immune dysregulation, and immune dysfunction, have raised concerns about safety (49–51). Compared with other approved PI3K-δ inhibitors, duvelisib has certain safety advantages. Compared with idelalisib, only inhibiting PI3K-δ, and based on preclinical data, duvelisib may reduce autoimmune complications through the inhibition of PI3K-γ (52, 53). Additionally, duvelisib doesn’t need infusion, without producing hyperglycemic effects mediated by PI3K-α isoform inhibition, which reduces the usage of copanlisib in older adults. A better understanding of the complexities of the adverse events and subgroup of lymphoid neoplasms patients benefitting most from duvelisib treatment could provide more precise treatment schedule. In this systematic review, we analyzed the efficacy and safety of duvelisib monotherapy in patients with relapsed or refractory lymphoid neoplasms. Besides, subgroup analysis was conducted to compare the efficacy and safety of duvelisib between different disease groups. These findings lead to offering evidence-based references for clinicians to optimize future clinical trials and treatment options.

## Methods

### Literature search

The study design and literature search strategy for this article followed Systematic Reviews and Meta-Analyses (PRISMA) guidelines. The relevant studies were identified by searching Medline (PubMed), Embase, Cochrane Library, and ClinicalTrials.gov. We used a combination of terms: “(Leukemia OR Lymphoma) AND ((Duvelisib) OR (COPIKTRA) OR (IPI-145))” to search for clinical studies evaluating the safety and efficacy of duvelisib in the treatment of relapsed or refractory lymphoid neoplasms, the data cut-off was May 20, 2022. There were no date or language restrictions.

### Inclusion criteria and exclusion criteria

Studies must meet the following inclusion criteria: 1) clinical trials in any phase of Duvelisib therapy for patients with relapsed/refractory CLL/SLL or relapsed/refractory NHL; 2) analyzable data on safety or efficacy available in the study; 3) drugs used in humans; 4) the patients are over 18 years old. Exclusion criteria are: (1) Studies not related to our topic; (2) studies without usable results; and (3) reviews, letters, editorials, patents, news, case reports, and retrospective or observed studies. Two authors independently searched, screened, and determined study eligibility, and any disagreements were resolved by discussion.

### Data extraction and quality control

Eligible studies were reviewed, and data were extracted independently by two authors. We identified the first author, publication year, ClinicalTrials.gov number, phase, study design and treatment, disease type, patients numbers, age, gender, previous systemic therapy, overall response rate (ORR), complete response rate (CR), partial response rate (PR), rate of stable disease (SDR), rate of progressive disease (PDR), median progression-free survival (mPFS), PFS, overall survival (OS), any grade AEs, grade ≥3 AEs, serious AEs, treatment-related discontinuation and death.

### Statistical analysis

The package meta version 5.2-0 Index in the R-4.1.1 was used to evaluate the results of efficacy and safety. To analyze heterogeneity between studies we included, the I-squared test (I2 test) was used. A random effects model was used when I2 >50%, and a fixed effect model was conducted when I2 ≤50%. All analyses were based on the intention-to-treat population of the studies included. The subgroup analysis by disease type or treatment was applied. Sensitivity analysis was carried out by using different effect models. No dose effect was considered. P < 0.05 suggested statistical significance.

### Study qualitative assessment

Methodological Indicators for Nonrandomized Studies (MINORS) were used to assess the methodological quality of non-randomized surgical studies included. MINORS contain 12 items; The first eight items are dedicated to non-comparative research. Items include the stated purpose of the study, The inclusion of consecutive patients, prospective collection of data, endpoints suitable for study purpose, unbiased endpoints evaluation, adequate follow-up time enough for the endpoint, loss to follow-up of patients not exceeding 5%, and sample size calculated prospectively. Each item is scored from 0 to 2; 0 is not reported, 1 is insufficiently reported, and 2 is adequately reported. The Cochrane Collaboration Risk of Bias Tool (Review Manager 5.4) is used to evaluate the bias of the RCT studies involved, including six criteria: random sequence generation, allocation concealment, blinding of participants and personnel, blinding of outcomes assessment, completeness of the outcome data, and selective reporting.

## Results

### Study selection

**Figure 1:**
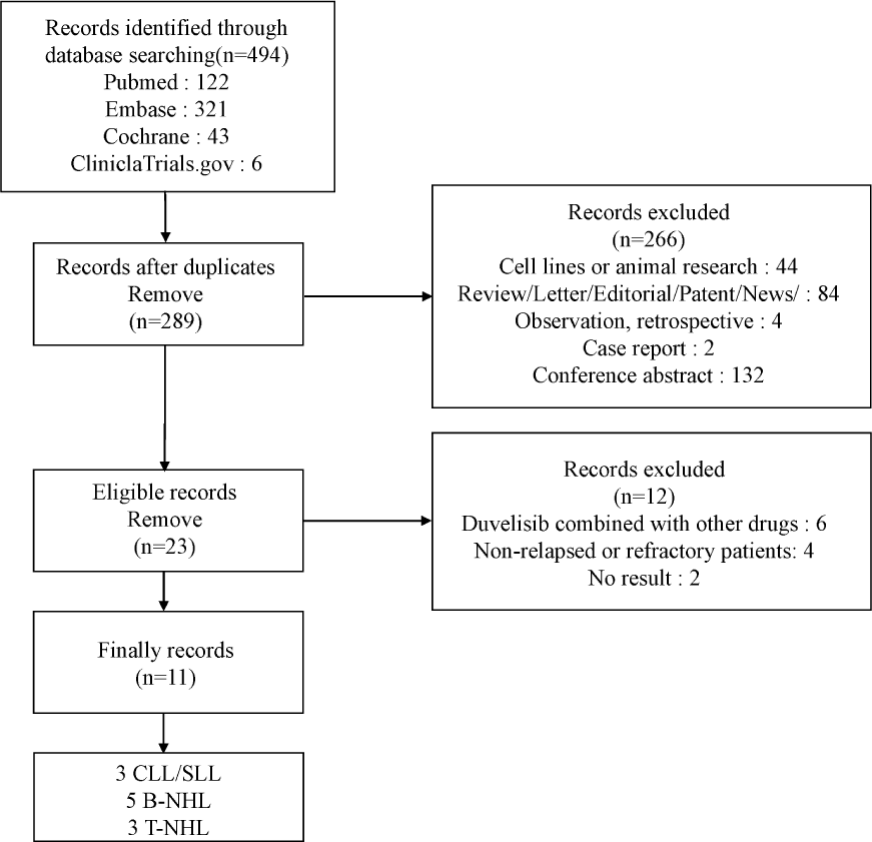
The flow chart.

Through the above search strategy, we retrieved 494 studies. 205 were dropped after the duplication check, and 266 were excluded for the reasons we mentioned above. After Inclusion, 6 studies were removed with combined therapy, 3 studies were removed with first-line treatment, and 2 studies had no analyzable results. **Figure 1** showed the details of our study selection process. Ultimately, our meta-analysis included 11 studies (23–33).

### Study characteristics

**Table 1** presents the baseline characteristics of the studies included. These studies were published from 2018 to 2022. We included total of 11 studies, 683 patients, of which 305 were CLL/SLL included in 3 studies, 378 in other 8 studies were NHL [187 B-cell indolent non-Hodgkin lymphoma(iNHL), 39 B-cell aggressive non-Hodgkin lymphoma (aNHL), 152 T-cell lymphomas (T-NHL)]. There were 6 phase I studies,3 phase II studies, and 2 phase III studies. Patients in all 11 studies received duvelisib monotherapy. We assessed safety and efficacy only in patients with RR CLL/SLL or RR NHL. All studies presented information on efficacy or safety.

**Table 1:** The characteristic of include studies.

Study	ClinicalTrials.govidentifier	Phase	Design	Disease	Treatment	No.ofpatients	Agerange	Male/female(n)	Median number ofprior regimen, (range)	ORR%	12-month PFS rate	12-month OS rate	mPFS(months)	Discontinued treatment due of Aes (n)	Any grade adverse events (n)	Grade≥3 adverse events (n)	Serious adverse events (n)
O’Brien-2018	NCT01476657	I	multicohort, single arm	relapsed or refractory CLL/SLL	25 or 75 mg BID in 28-day cycles	55	66(42–82)	42/13	4(1–11)	56.4%	57.1%	65.5%	ALL:15.7 TP53 mutation/17p-deletion:27.9	20	_	_	_
Flinn-2018	NCT02004522	III	multicenter, randomized	relapsed or refractory CLL/SLL	25 mg BID in 28-day cycles	160	69(39-90)	96/64	2(1-10)	73.8%	60.0%	86.3%	ALL:13.3 TP53 mutation/17p-deletion:12.7	55	156	138	115
Davids-2020	NCT02049515	III	two-arm, non-randomized	relapsed or refractory CLL/SLL	25 mg BID in 28-day cycles	90	68(39–90)	57/33	3(2-8)	76.7%	64.4%	82.2%	ALL:15.7 TP53 mutation/17p-deletion:14.7	47	90	80	67
Flinn-2018	NCT01476657	I	multicohort, single arm	relapsed or refractory idolent NHL	25 or 75 mg BID in 28-day cycles	31	64(37-78)	18/13	3 (1-8)	58.1%	58.1%	77.4%	9.5	6	_	_	_
Flinn-2019	NCT01882803	II	single arm	relapsed or refractory idolent NHL	25 mg BID in 28-day cycles	129	65(30-90)	88/41	3 (1-18)	47.3%	31.8%	76.7%	14.7	40	128	114	74
Zheng-2021	NCT04707079	II	multicenter, single arm	relapsed or refractory Follicular lymphoma	25 mg BID in 28-day cycles	23	49(31-70)	16/7	≥2	82.6%	_	_		_	21	12	_
Izutsu-2020	NCT02598570	I	multicenter, single arm	relapsed or refractory B cell NHL	25 mg BID in 28-day cycles	7	61(54–74)	4/3	3 (1–5)	71.4%	_	_		2	7	_	2
Flinn-2018	NCT01476657	I	multicohort, single arm	relapsed or refractory B cell NHL	25 or 75 mg BID in 28-day cycles	36	_	_	≥1	27.8%	_	_		_	_	_	_
Horwitz-2018	NCT01476657	I	multicohort, single arm	relapsed or refractory T cell NHL	25 or 75 mg BID in 28-day cycles	35	64 (34-86)	16/19	4 (1-10)	40.0%	31.4%	65.7%	PTCL:8.3 CTCL:4.5	13	34	24	20
Horwitz-2019	NCT02783625	I	single arm	relapsed or refractory T cell NHL	25 or 75 mg BID in 1 month lead-in	16	_	_	≥1	50.0%	_	_		_	_	_	_
Zinzani-2022	NCT03372057	II	multicenter, parallel cohort	relapsed or refractory T cell NHL	25 mg BID after 75 mg BID for 2 cycles	101	67(21-92)	_	3 (1–9)	48.5%	_	_	3.6	20	_	_	_

### Assessment of study quality

The MINORS scores of 10 single arm studies ranged from 7 to 13. 3 studies without full context cannot be evaluated totally. The item of sample size calculated prospectively was not mentioned. The bias of the only 1 RCT study included was assessed by the Cochrane Collaboration Risk of Bias Tool with an acceptable quality result. Therefore, the overall quality of the 11 studies included was satisfactory. More details are shown in **Supplementary Figure 1** and **Supplementary Table 1**.

### Efficacy

All studies reported the efficacy outcomes such as overall response rate (ORR), complete response rate (CR), partial response rate (PR), stable disease rate (SDR), and progressive disease rate (PDR). The response was based on International Workshop on CLL or Revised International Working Group response criteria for CLL/SLL patients, and the International Working Group response criteria for NHL patients.

**Figure 2:**
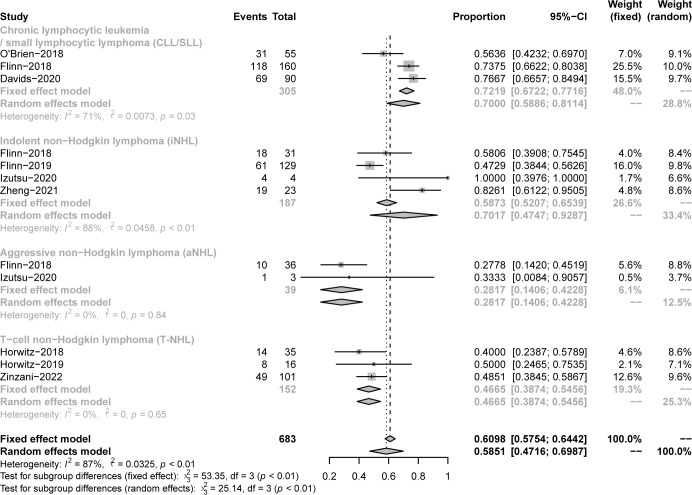
The forest plot of pooled ORR. Disease including chronic lymphocytic leukemia/small lymphocytic lymphoma (CLL/SLL), indolent non−Hodgkin lymphoma (iNHL), aggressive non−Hodgkin lymphoma (aNHL) and T−cell non−Hodgkin lymphoma (T-NHL).

**Figure 3:**
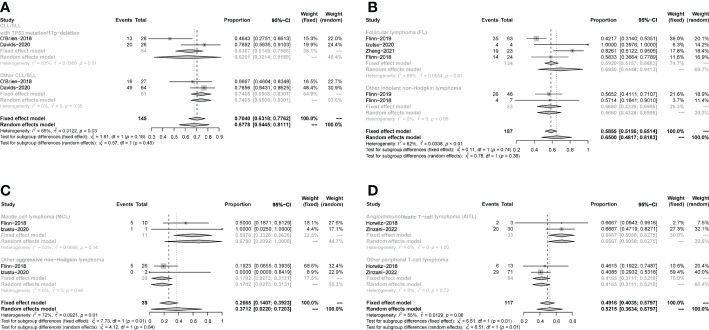
Subgroup analysis of the pooled ORR. **(A)**. Subgroup of CLL/SLL, the pooled ORR of CLL/SLL with TP53 mutation/17p−deletion and others. **(B)**. Subgroup of iNHL, the pooled ORR of Follicular lymphoma (FL) and others. **(C)**. Subgroup of aNHL, the pooled ORR of MCL and others. **(D)**. Subgroup of PTCL, the pooled ORR of AITL and others.

All studies focused on evaluating the ORR. The pooled ORR in CLL/SLL, iNHL, B-aNHL and T-NHL was 70% (59-81%), 70% (48-93%), 28% (14-42%) and 47% (39-55%), respectively (**Figure 2**). Additionally, the ORR in CLL/SLL patients with or without TP53 mutation/17p-deletion (62% vs. 74%, p=0.45) had no significant difference (**Figure 3A**). Follicular lymphoma (FL) had higher ORR than iNHL, yet without statistical significance (69% vs. 57%, p=0.38) (**Figure 3B**). Besides, Mantle cell lymphoma (MCL) patients had higher pooled ORR than other aNHL (68% vs. 17%, p=0.04) (**Figure 3C**). The ORR was 49% and 32% in PTCL and CTCL, respectively (**Supplementary Figure 2A**). In the subgroup of PTCL, Angioimmunoblastic T cell Lymphoma (AITL) patients had higher pooled ORR than other PTCL patients (67% vs. 42%, p=0.01) (**Figure 3D**).

**Figure 4:**
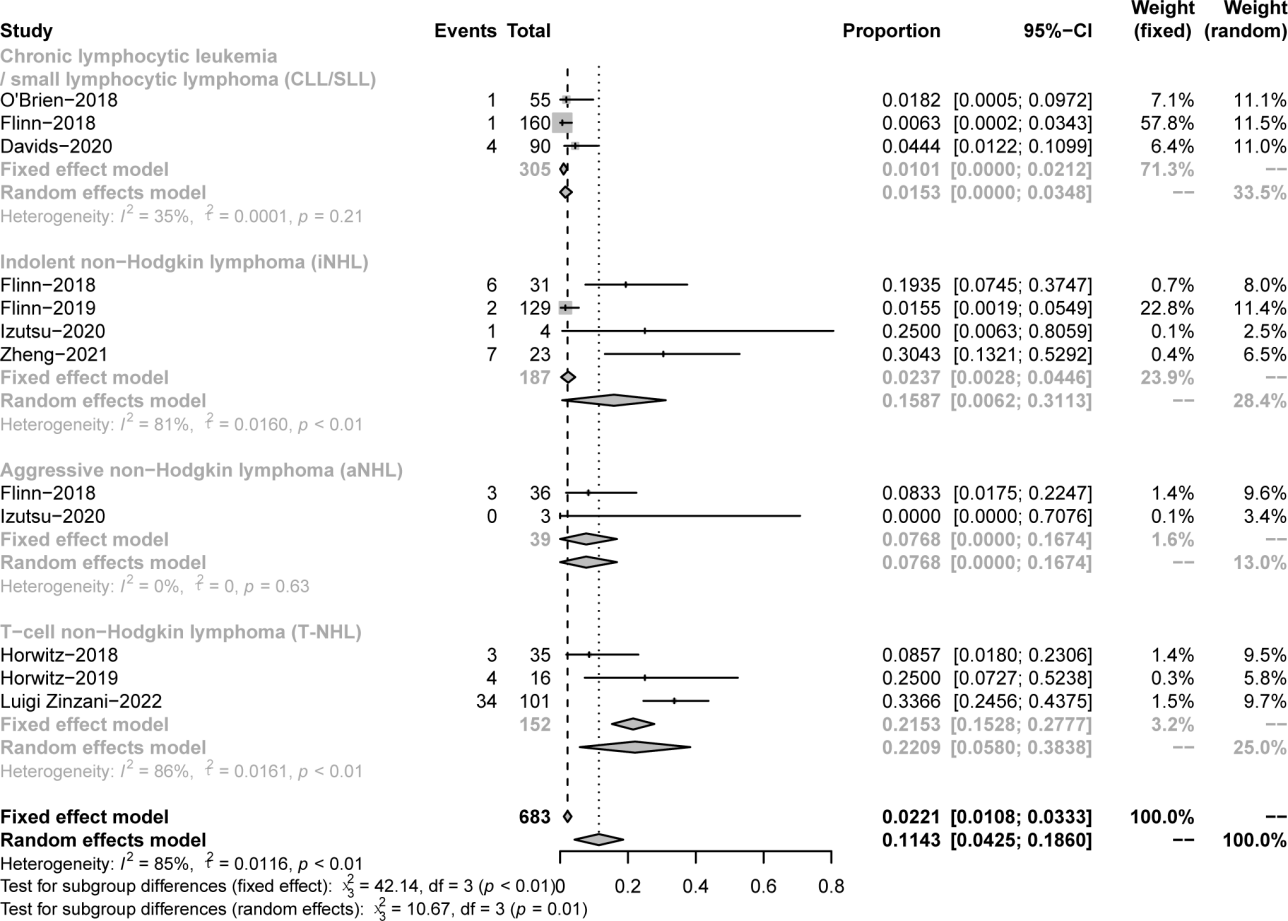
The forest plot of pooled CR. Disease includes chronic lymphocytic leukemia/small lymphocytic lymphoma (CLL/SLL), indolent non−Hodgkin lymphoma (iNHL), aggressive non−Hodgkin lymphoma (aNHL) and T−cell non−Hodgkin lymphoma (T-NHL).

**Figure 5:**
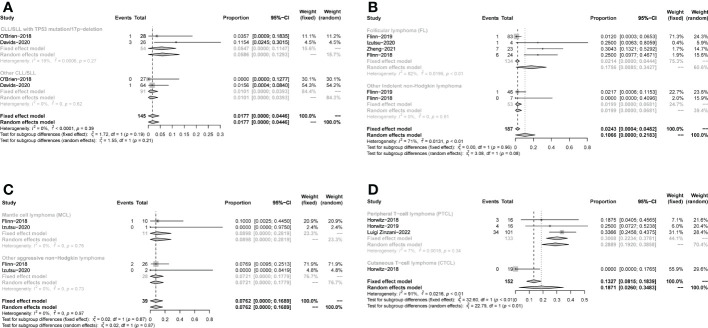
Subgroup analysis of the pooled CR. **(A)**. Subgroup of CLL/SLL, the pooled CR of CLL/SLL with TP53 mutation/17p−deletion and others. **(B)**. Subgroup of iNHL, the pooled CR of Follicular lymphoma (FL) and others. **(C)**. Subgroup of aNHL, the pooled CR of MCL and others. **(D)**. Subgroup of T-NHL, the pooled CR of PTCL and CTCL.

The pooled CR in CLL/SLL, iNHL, B-aNHL and T-NHL was 2% (0–1%), 16% (1–31%), 8% (0–17%) and 22% (6–38%), respectively (**Figure 4**). In the subgroup analysis, the CR in CLL/SLL patients with or without TP53 mutation/17p-deletion (6% vs. 1%, p=0.21) had no significant difference (**Figure 5A**). FL had higher CR than other iNHL, yet without statistical significance (18% vs. 2%, p=0.08) (**Figure 5B**). Besides, the CR in MCL patients and CR in other aNHL are similar (9% vs. 7%, p=0.87) (**Figure 5C**). PTCL had a higher CR than CTCL (29% vs. 0%, p<0.01) (**Figure 5D**).

**Figure 6:**
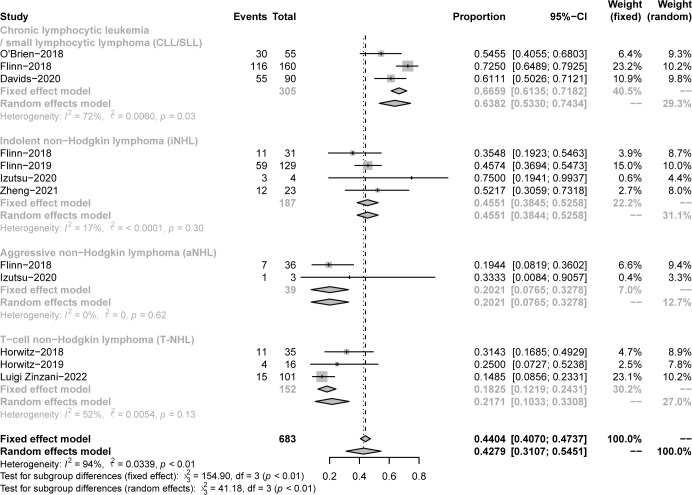
The forest plot of pooled PR, disease includes Chronic lymphocytic leukemia/small lymphocytic lymphoma (CLL/SLL), Indolent non−Hodgkin lymphoma (iNHL), Aggressive non−Hodgkin lymphoma (aNHL) and T−cell non−Hodgkin lymphoma (T-NHL).

**Figure 7:**
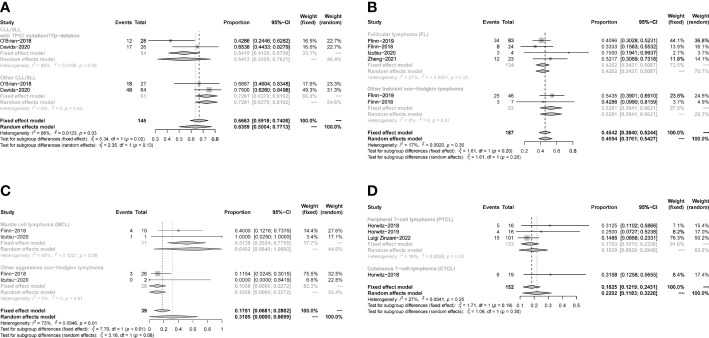
The forest plot of pooled PR of disease subgroup, **(A)**. Subgroup of CLL/SLL, the pooled PR of CLL/SLL with TP53 mutation/17p−deletion and others. **(B)**. Subgroup of iNHL, the pooled PR of Follicular lymphoma (FL) and others. **(C)**. Subgroup of aNHL, the pooled PR of MCL and others. **(D)**. Subgroup of T-NHL, the pooled PR of PTCL and CTCL.

The pooled PR in CLL/SLL, iNHL, B-aNHL and T-NHL was 64% (53–74%), 46% (39–53%), 20% (8–33%) and 22% (10–33%), respectively (**Figure 6**). In the subgroup analysis, the PR in CLL/SLL patients with or without TP53 mutation/17p-deletion (54% vs. 73%, p=0.13) had no significant difference (**Figure 7A**). the PR in FL and that in other iNHL had no significant difference (43% vs. 53%, p=0.20) (**Figure 7B**). Besides, the PR in MCL had higher PR than other aNHL, yet without statistical significance (64% vs. 11%, p=0.08) (**Figure 7C**). There is no significant difference between the PR of PTCL and CTCL (19% vs. 32%, p=0.30) (**Figure 7D**).

The pooled SDR in CLL/SLL, iNHL, and T-NHL was 22% (12–33%), 25% (8–42%), and 10% (0–27%), respectively (**Supplementary Figure 3A**). The pooled PDR in CLL/SLL, iNHL, and T-NHL was 1% (0–3%), 11% (6–16%), 43% (29–57%), respectively (**Supplementary Figure 3B**). In the subgroup of T-NHL, the SDR was 2% (0–5%) and 32% (13–57%) in PTCL and CTCL, respectively (**Supplementary Figure 2B**). The PDR was 47% (38–56%) and 32% (13–57%) in PTCL and CTCL, respectively (**Supplementary Figure 2C**). No enough SDR or PDR data is available to analyze other subgroups.

**Figure 8:**
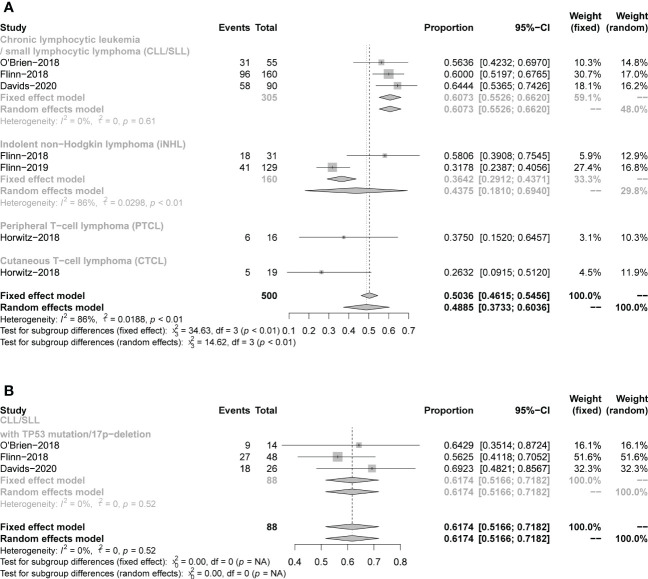
The forest plot of pooled 12-month PFS rate, **(A)**. pooled 12-month PFS rate of CLL/SLL, iNHL, PTCL and CTCL. **(B)**. Pooled 12-month PFS rate of CLL/SLL with TP53 mutation/17p-deletion.

**Figure 9:**
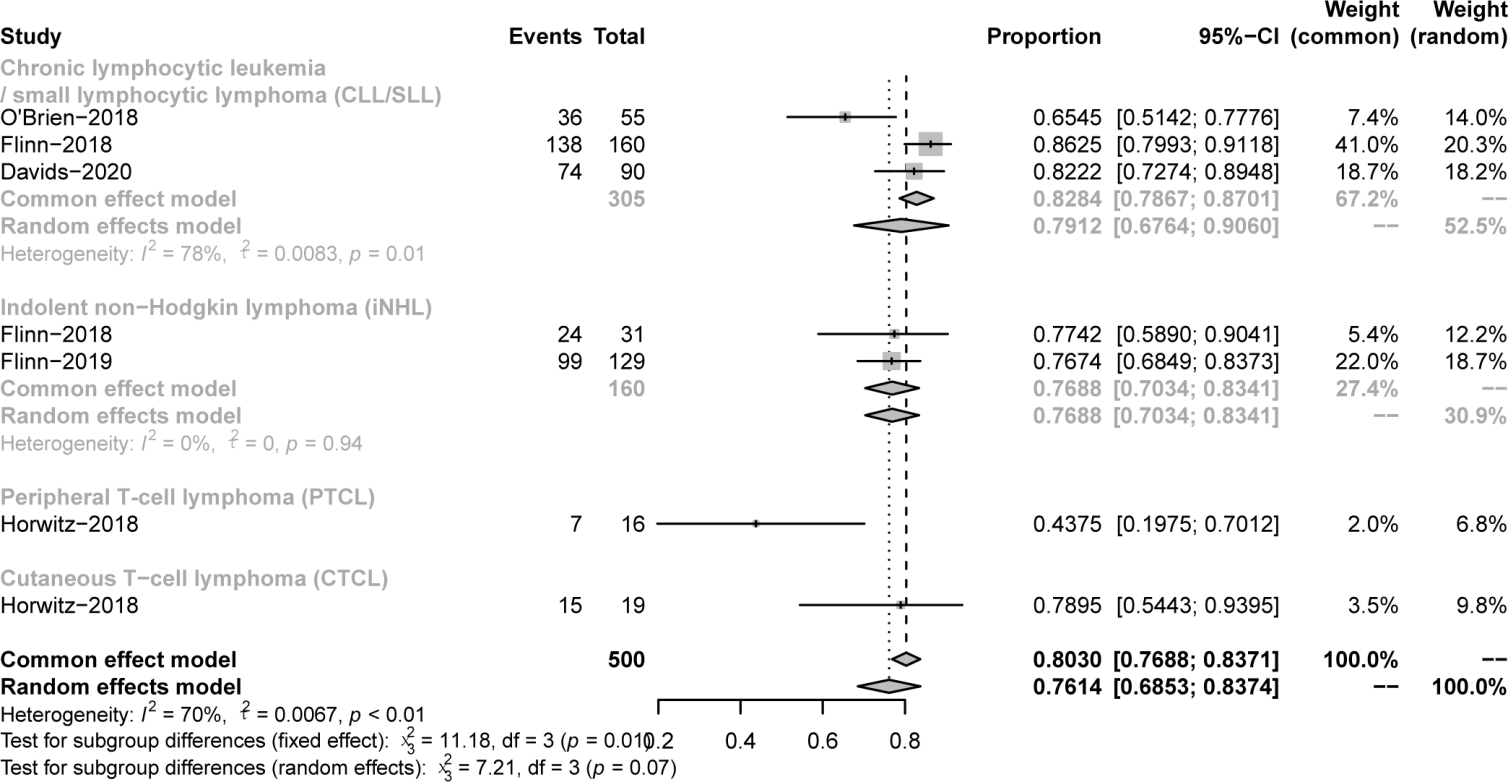
The forest plot of pooled 12-month OS rate of CLL/SLL, iNHL, PTCL and CTCL.

Seven studies presented mPFS data. For all CLL/SLL patients, the mPFS reported in the three studies was 15.7, 13.3, and 15.7 months, respectively. For CLL/SLL patients with TP53 mutation/17p−deletion, the mPFS was 27.9, 12.7, and 14.7, respectively. For patients with iNHL, the mPFS reported in the two studies was 9.5 and 14.7 months, respectively. The mPFS for patients with PTCL in the reported 2 studies was 8.3, and 3.6 months, and CTCL in 1 study was 4.5 months. The mPFS data reported in studies were shown in Table1. The rate of PFS and OS was performed to assess the efficacy of duvelisib treatment in CLL/SLL and NHL. For all patients, the pooled 12-month PFS rate was 49% (37%−60%). Besides, in CLL/SLL, iNHL, and CLL/SLL with TP53 mutation/17p−deletion, the pooled 12-month PFS rate was 61% (55–66%),44% (18–69%), and 62% (52–72%), respectively (**Figure 8**). Only 1 T-NHL study reported the 12-month PFS of PTCL and CTCL was 38% (15–65%), 26% (9–51%), respectively. Moreover, the 24-month PFS rate was also similar to the rate between all CLL/SLL patients (27%, 22–32%) and those with TP53 mutation/17p−deletion (27%, 12–42%) (**Supplementary Figure 4**). For all patients, the pooled 12-month OS rate was 76% (69−84%). Besides, in CLL/SLL and iNHL, the pooled 12-month OS rate was 79% (68–66%) and 77% (70–83%), respectively (**Figure 9**). Only 1 T-NHL study reported the 12-month OS of PTCL and CTCL was 44% (20–70%) and 79% (54–94%), respectively.

### Safety

**Figure 10:**
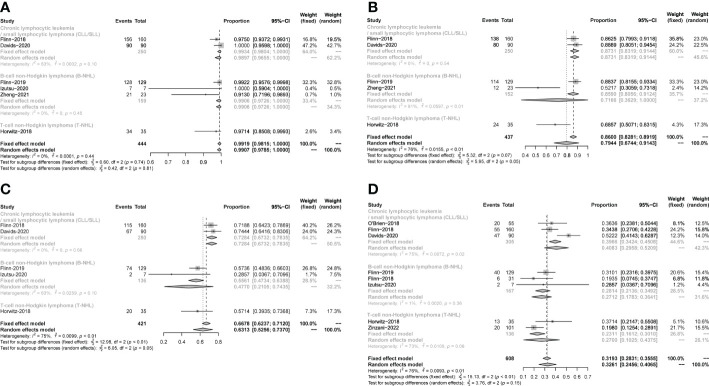
The forest plot of incidences of AEs. **(A)**. AEs in any grade. **(B)**. AEs in grade ≥3. **(C)**. Serious AEs. **(D)**. Treatment discontinuation due to AEs.

The safety profiles in individual disease subgroups were generally consistent with those of the entire subjects. The safety assessments recorded in studies on patients were listed in **Table 1**. Six studies were included in the analysis of the pooled incidence of any grade AEs (99%, 98−100%), and five studies were included to evaluate the pooled incidence of grade ≥3 AEs (79%,67−91%). 5 studies were included in the analysis of pooled serious AEs (63%,53–74%). The pooled rate of treatment discontinuation due to AEs was 33% (25–41%) in 8 included studies (**Figure 10**). And the most frequent AEs leading to treatment discontinuation were elevated transaminases (6%, 0-11%), colitis (5%, 2-7%), diarrhea (4%, 2-6%) (**Supplementary Figure 5**).

The most frequent any-grade AEs were Diarrhea (47%, 43-52%), ALT/AST increased (39%, 23-55%), neutropenia (38%, 26-50%), fatigue (29%, 18-40%), fever (29%, 25-32%) and cough (26%, 18-34%). The most frequent grade 3 or greater AEs were neutropenia (25%, 19-31%), ALT/AST increased (16%, 5-26%), diarrhea (12%, 6-18%) and anemia (12%, 5 -18%) (**Table 2**).

**Table 2:** The incidence of adverse events in all grade or grade equal or greater than 3.

AEs	Diease	Any grade						Grade≥3					
		Includestudy	Event	Total	Pooled rate(95%CI)	Model	P-value	Includestudy	Event	Total	Pooled rate(95%CI)	Model	P-value
Hematological													
Thrombocytopenia	CLL/SLL	3	45	305	0.1461 [0.0837; 0.2084]	Random		3	27	305	0.0881 [0.0322; 0.1440]	Random	
	B-NHL	4	39	190	0.2005 [0.1439; 0.2571]	Fixed		3	19	167	0.1048 [0.0586; 0.1509]	Fixed	
	T-NHL	–	–	–	–	–		–	–	–	–	–	
	ALL	7	84	495	0.1576 [0.1258; 0.1893]	Fixed		6	46	472	0.0847 [0.0598; 0.1096]	Fixed	
Anemia	CLL/SLL	3	62	305	0.2082 [0.0595; 0.3569]	Random		3	34	305	0.1124 [0.0048; 0.2199]	Random	
	B-NHL	2	40	160	0.2475 [0.1807; 0.3142]	Fixed		2	23	160	0.1434 [0.0891; 0.1977]	Fixed	
	T-NHL	–	–	–	–	–		–	–	–	–	–	
	ALL	5	102	465	0.2145 [0.1257; 0.3033]	Random		5	57	465	0.1186 [0.0533; 0.1840]	Random	
Neutropenia	CLL/SLL	3	106	305	0.3752 [0.1993; 0.5510]	Random		3	93	305	0.3133 [0.2081; 0.4186]	Random	
	B-NHL	4	68	190	0.4402 [0.2492; 0.6312]	Random		4	54	232	0.2350 [0.1382; 0.3319]	Fixed	
	T-NHL	1	7	35	0.2000 [0.0844; 0.3694]	–		2	25	136	0.1836 [0.1185; 0.2486]	Fixed	
	ALL	8	181	530	0.3795 [0.2643; 0.4947]	Random		9	172	673	0.2508 [0.1912; 0.3105]	Random	
Febrile neutropenia	CLL/SLL	1	9	55	0.1641 [0.0661; 0.2610]			1	9	55	0.1641 [0.0661; 0.2610]		
	B-NHL	1	12	129	0.0930 [0.0434; 0.1431]			1	12	129	0.0930 [0.0434; 0.1431]		
	ALL	2	21	184	0.1152 [0.0511; 0.1798]	Fixed		2	21	184	0.1152 [0.0511; 0.1798]	Fixed	
Non-Hematological													
ALL infections	CLL/SLL	2	150	215	0.6983 [0.6370; 0.7596]	Fixed		1	29	90	0.3222 [0.2275; 0.4290]		
	B-NHL	1	19	31	0.6129 [0.4219; 0.7815]			2	12	96	0.1463 [0.0000; 0.3371]	Random	
	T-NHL	–	–	–	–	–		2	20	136	0.1791 [0.0000; 0.3602]	Random	
	ALL	3	169	246	0.6886 [0.6309; 0.7463]	Fixed		5	61	322	0.1941 [0.0852; 0.3030]	Random	
Fatigue	CLL/SLL	2	41	215	0.2464 [0.0000; 0.4977]	Random		2	8	215	0.0520 [0.0000; 0.1451]	Random	
	B-NHL	2	49	160	0.3268 [0.1965; 0.4570]	Random		2	6	160	0.0245 [0.0000; 0.0700]	Random	
	T-NHL	1	11	35	0.3143 [0.1685; 0.4929]			1	3	35	0.0857 [0.0180; 0.2306]		
	ALL	5	101	410	0.2885 [0.1805; 0.3966]	Random		5	17	410	0.0348 [0.0024; 0.0672]	Random	
Dyspnoea	CLL/SLL	2	29	215	0.1582 [0.0260; 0.2904]	Random		2	7	215	0.0291 [0.0067; 0.0516]	Fixed	
	B-NHL	1	7	31	0.2258 [0.0959; 0.4110]			1	0	31	0.0000 [0.0000; 0.1122]		
	T-NHL	1	6	35	0.1714 [0.0656; 0.3365]			1	4	35	0.1143 [0.0320; 0.2674]		
	ALL	4	42	281	0.1671 [0.0917; 0.2424]	Fixed		4	11	281	0.0256 [0.0052; 0.0461]	Fixed	
Cough	CLL/SLL	3	64	305	0.2182 [0.1064; 0.3300]	Random		3	2	305	0.0039 [0.0000; 0.0146]	Fixed	
	B-NHL	2	47	160	0.2906 [0.2206; 0.3607]	Fixed		2	0	160	0.0000 [0.0000; 0.0103]	Fixed	
	T-NHL	1	12	35	0.3429 [0.1913; 0.5221]			1	0	35	0.0000 [0.0000; 0.1000]		
	ALL	6	123	500	0.2620 [0.1844; 0.3396]	Random		6	2	500	0.0018 [0.0000; 0.0091]	Fixed	
Fever	CLL/SLL	3	84	305	0.2742 [0.2242; 0.3242]	Fixed		3	10	305	0.0308 [0.0114; 0.0501]	Fixed	
	B-NHL	2	48	160	0.3698 [0.1082; 0.6314]	Random		2	1	160	0.0007 [0.0000; 0.0112]	Fixed	
	T-NHL	1	13	35	0.3714 [0.2147; 0.5508]			1	0	35	0.0000 [0.0000; 0.1000]		
	ALL	6	145	500	0.2848 [0.2456; 0.3240]	Fixed		6	11	500	0.0066 [0.0000; 0.0157]	Fixed	
Diarrhea	CLL/SLL	3	148	305	0.4852 [0.4292; 0.5413]	Fixed		3	49	305	0.1528 [0.0781; 0.2275]	Random	
	B-NHL	3	82	167	0.4892 [0.4138; 0.5646]	Fixed		3	28	167	0.1615 [0.1060; 0.2170]	Fixed	p<0.01, B-NHL vs T-NHL
	T-NHL	1	11	35	0.3143 [0.1685; 0.4929]			2	7	136	0.0326 [0.0000; 0.1004]	Random	p=0.02, CLL/SLL vs T-NHL
	ALL	7	241	507	0.4730 [0.4299; 0.5162]	Fixed		8	84	608	0.1232 [0.0623; 0.1841]	Fixed	
Nausea	CLL/SLL	3	60	305	0.1892 [0.0921; 0.2863]	Random		3	1	305	0.0006 [0.0000; 0.0079]	Fixed	
	B-NHL	3	51	167	0.2988 [0.2299; 0.3677]	Fixed		3	4	167	0.0134 [0.0000; 0.0329]	Fixed	
	T-NHL	1	9	35	0.2571 [0.1249; 0.4326]			1	0	35	0.0000 [0.0000; 0.1000]		
	ALL	7	120	507	0.2336 [0.1623; 0.3049]	Random		7	5	507	0.0022 [0.0000; 0.0090]	Fixed	
Vomiting	CLL/SLL	3	43	305	0.1377 [0.0991; 0.1763]	Fixed		3	1	305	0.0006 [0.0000; 0.0079]	Fixed	
	B-NHL	3	31	167	0.1852 [0.1263; 0.2441]	Fixed		3	6	167	0.0363 [0.0056; 0.0671]	Fixed	
	ALL	6	74	472	0.1519 [0.1197; 0.1842]	Fixed		6	7	472	0.0025 [0.0000; 0.0097]	Fixed	
Decreased appetite	CLL/SLL	3	43	305	0.1457 [0.0694; 0.2221]	Fixed		3	1	305	0.0006 [0.0000; 0.0079]	Fixed	
	B-NHL	2	26	160	0.1588 [0.1024; 0.2153]	Fixed		2	1	160	0.0066 [0.0000; 0.0234]	Fixed	
	ALL	5	69	465	0.1385 [0.1073; 0.1696]	Fixed		5	2	465	0.0015 [0.0000; 0.0083]	Fixed	
Hypokalemia	CLL/SLL	1	10	55	0.1821 [0.0801; 0.2840]			1	3	55	0.0551 [0.0008; 0.1151]		
	B-NHL	1	17	129	0.1320 [0.0734; 0.1901]			1	4	129	0.0314 [0.0001; 0.0608]		
	ALL	2	27	184	0.1442 [0.0931; 0.1948]	Fixed		2	7	184	0.0358 [0.0091; 0.0623]	Fixed	
URTI	CLL/SLL	2	40	215	0.2025 [0.0908; 0.3143]	Random		2	1	215	0.0007 [0.0000; 0.0091]	Fixed	
	B-NHL	1	6	31	0.1935 [0.0745; 0.3747]			1	1	31	0.0323 [0.0008; 0.1670]		
	ALL	3	46	246	0.1798 [0.1321; 0.2274]	Fixed		3	2	246	0.0011 [0.0000; 0.0095]	Fixed	
Arthralgia	CLL/SLL	1	14	55	0.2545 [0.1467; 0.3900]			1	0	55	0.0000 [0.0000; 0.0649]		
	B-NHL	2	20	136	0.1471 [0.0875; 0.2066]	Fixed		2	0	136	0.0000 [0.0000; 0.0268]	Fixed	
	ALL	3	34	191	0.1697 [0.1169; 0.2226]	Fixed		3	0	191	0.0000 [0.0000; 0.0191]	Fixed	
Edema	CLL/SLL	1	10	55	0.1818 [0.0908; 0.3090]			1	0	55	0.0000 [0.0000; 0.0649]		
	B-NHL	2	27	160	0.1687 [0.1107; 0.2267]	Fixed		2	4	160	0.0164 [0.0000; 0.0397]	Fixed	
	ALL	3	37	215	0.1719 [0.1215; 0.2223]	Fixed		3	3	215	0.0086 [0.0000; 0.0256]	Fixed	
Pneumonia	CLL/SLL	3	62	305	0.2226 [0.0838; 0.3614]	Random		3	45	305	0.1399 [0.1012; 0.1786]	Fixed	
	B-NHL	2	11	136	0.0795 [0.0341; 0.1250]	Fixed	p=0.04 (CLL/SLL vs B-NHL)	2	8	136	0.0562 [0.0176; 0.0949]	Fixed	p<0.01 (CLL/SLL vs B-NHL)
	T-NHL	1	8	35	0.2286 [0.1042; 0.4014]			2	8	136	0.0829 [0.0000; 0.2293]	Random	
	ALL	7	81	476	0.1839 [0.0970; 0.2708]	Random		8	64	608	0.1026 [0.0549; 0.1502]	Random	
Stomatitis	CLL/SLL	1	10	55	0.1818 [0.0908; 0.3090]			1	3	55	0.0545 [0.0114; 0.1512]		
	B-NHL	2	7	38	0.1775 [0.0567; 0.2982]	Fixed		2	1	38	0.0035 [0.0000; 0.0460]	Fixed	
	ALL	3	17	93	0.1800 [0.1021; 0.2579]	Fixed		3	4	93	0.0193 [0.0000; 0.0546]	Fixed	
Colitis	CLL/SLL	2	33	250	0.1320 [0.0900; 0.1740]	Fixed		2	29	250	0.1159 [0.0762; 0.1556]	Fixed	
	B-NHL	3	14	167	0.0777 [0.0373; 0.1181]	Fixed		2	8	136	0.0562 [0.0176; 0.0949]	Fixed	p=0.03, CLL/SLL vs B-NHL
	T-NHL	1	1	101	0.0099 [0.0003; 0.0539]		p<0.01, CLL/SLL vs T-NHL	1	0	101	0.0000 [0.0000; 0.0359]		p<0.01, CLL/SLL vs T-NHL
	ALL	6	48	512	0.0788 [0.0287; 0.1288]	Random		5	36	487	0.0660 [0.0091; 0.1229]	Random	
Constipation	CLL/SLL	1	26	160	0.1625 [0.1090; 0.2290]			1	1	160	0.0063 [0.0002; 0.0343]		
	B-NHL	2	16	136	0.1174 [0.0633; 0.1715]	Fixed		2	0	136	0.0000 [0.0000; 0.0106]	Fixed	
	T-NHL	1	6	35	0.1714 [0.0656; 0.3365]			1	0	35	0.0000 [0.0000; 0.1000]		
	ALL	4	48	331	0.1417 [0.1042; 0.1792]	Fixed		4	1	331	0.0020 [0.0000; 0.0104]	Fixed	
Asthenia	CLL/SLL	2	29	250	0.1158 [0.0762; 0.1555]	Fixed		2	3	250	0.0059 [0.0000; 0.0185]	Fixed	
	B-NHL	1	15	129	0.1163 [0.0666; 0.1845]			1	3	129	0.0088 [0.0000; 0.0203]		
	ALL	3	44	379	0.1160 [0.0838; 0.1482]	Fixed		3	6	379	0.0110 [0.0000; 0.0266]	Fixed	
Abdominal pain	CLL/SLL	2	26	250	0.1038 [0.0660; 0.1416]	Fixed		2	4	250	0.0150 [0.0000; 0.0301]	Fixed	
	B-NHL	1	19	129	0.1473 [0.0911; 0.2204]			1	2	129	0.0155 [0.0019; 0.0549]		
	ALL	3	45	379	0.1158 [0.0836; 0.1479]	Fixed		3	6	379	0.0152 [0.0029; 0.0275]	Fixed	
Bronchitis	CLL/SLL	1	21	160	0.1262 [0.0750; 0.1763]			1	5	160	0.0312 [0.0102; 0.0714]		
	B-NHL	1	1	7	0.1428 [0.0000; 0.4021]			1	0	7	0.0000 [0.0000; 0.4096]		
	ALL	2	22	167	0.1263 [0.0768; 0.1763]	Fixed		2	5	167	0.0304 [0.0027; 0.0581]	Fixed	
Headache	B-NHL	2	28	160	0.1696 [0.1117; 0.2275]	Fixed		2	1	160	0.0007 [0.0000; 0.0112]	Fixed	
	T-NHL	1	8	35	0.2286 [0.1042; 0.4014]			1	0	35	0.0000 [0.0000; 0.1000]		
	ALL	3	36	195	0.1783 [0.1249; 0.2317]			3	1	195	0.0006 [0.0000; 0.0108]	Fixed	
ALT/AST increased	CLL/SLL	1	17	55	0.3091 [0.1914; 0.4481]			3	13	305	0.0319 [0.0123; 0.0515]		
	B-NHL	4	49	190	0.3657 [0.1489; 0.5825]	Random		3	20	167	0.1863 [0.0000; 0.3960]	Random	
	T-NHL	1	20	35	0.5714 [0.3935; 0.7368]			2	37	136	0.2990 [0.1327; 0.4653]	Random	p<0.01 (CLL/SLL vs T-NHL)
	ALL	6	86	280	0.3890 [0.2331; 0.5449]	Random		8	70	608	0.1562 [0.0540; 0.2583]	Random	
Rash	CLL/SLL	2	37	250	0.1613 [0.0311; 0.2916]	Random		2	7	250	0.0238 [0.0049; 0.0426]	Fixed	
	B-NHL	4	44	190	0.1933 [0.1372; 0.2493]	Fixed		3	14	167	0.0257 [0.0000; 0.0535]	Fixed	
	ALL	7	91	523	0.1752 [0.1165; 0.2339]	Random		5	21	417	0.0246 [0.0083; 0.0409]	Fixed	
Rash maculo-papular	CLL/SLL	1	10	55	0.1818 [0.0908; 0.3090]			1	0	55	0.0000 [0.0000; 0.0649]		
	B-NHL	2	7	38	0.1822 [0.0597; 0.3048]	Fixed		2	2	38	0.0494 [0.0000; 0.1307]	Fixed	
	T-NHL	1	8	35	0.2286 [0.1042; 0.4014]			1	6	35	0.1714 [0.0656; 0.3365]		
	ALL	4	25	128	0.1932 [0.1249; 0.2615]	Fixed		4	8	128	0.0501 [0.0000; 0.1253]	Random	
Pneumonitis	CLL/SLL	1	5	55	0.0909 [0.0302; 0.1995]			2	23	250	0.0804 [0.0076; 0.1531]	Random	
	B-NHL	1	2	31	0.0645 [0.0079; 0.2142]			3	12	167	0.0705 [0.0299; 0.1111]	Fixed	
	T-NHL	–	–	–	–	–		1	1	101	0.0099 [0.0003; 0.0539]		
	ALL	2	7	86	0.0794 [0.0223; 0.1365]	Fixed		6	36	518	0.0565 [0.0179; 0.0951]	Random	

The subgroup analysis of CLL/SLL, B-NHL, and T-NHL were shown in the **Table 2**. The incidence of grade≥3 transaminase elevations in CLL/SLL (3%) is much lower than in B-NHL (19%, *p*=0.05) or T-NHL (30%, *p* < 0.01). And the incidence of grade≥3 diarrhea in T-NHL (3%) is much lower than in CLL/SLL (15%, *p*=0.02) and B-NHL (16%, p<0.01). Furthermore, the incidence of grade≥3 colitis in CLL/SLL(12%) is higher than in T-NHL(0%,*p* < 0.01) and B-NHL (6%, p=0.03) (**Table 2**). Consistent with this difference, elevated transaminases (CLL/SLL:1%, B-NHL:8%, T-NHL:15%) was a major factor for treatment discontinuation in NHL, while diarrhea (CLL/SLL:5%, B-NHL:2%) or colitis (CLL/SLL:6%, B-NHL:2%, T-NHL: 6%) were major factors for treatment discontinuation in CLL/SLL (**Supplementary Figure 5**). Furthermore, the CLL/SLL patients have shown higher incidence of any grade (22% vs. 8%, p=0.04) and grade≥3 pneumonia (14% vs. 6%, p<0.01) compared to B-NHL patients (**Table 2**).

**Figure 11:**
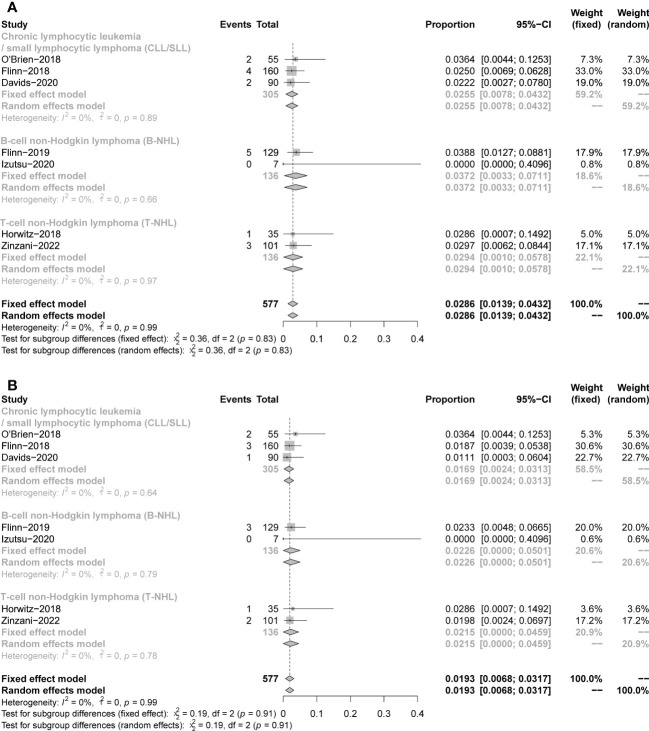
The forest plot of treatment-related mortality. **(A)**. The incidence of fatal AEs related to duvelisib. **(B)**. The incidence of fatal infectious AEs related to duvelisib.

A total of 17 fatal AEs related to duvelisib application by 577 patients were reported in the 7 studies we included (pooled rate of 3%). Infectious complications are the leading causes of duvelisib-related mortality (n=12, pooled rate of 2%). Details were shown in **Table 3** and **Figure 11**.

**Table 3:** AEs leading to death related to treatment.

	Total patients	number	AEs leading to death related to treatment(n)
O’Brien-2018	55	2	respiratory syncytial viral pneumonia (1), metabolic acidosis in the setting of sepsis and renal failure (1)
Flinn-2018	160	4	staphylococcal pneumonia (2), sepsis (1), general health deterioration (1)
Davids-2020	90	2	general health deterioration (1), Pneumocystis jirovecii pneumonia (1)
Izutsu-2020	7	0	-
Flinn-2019	129	5	severe skin toxicity (2), suspected viral infection (1), fatal septic shock (1), pancolitis (1)
Horwits-2018	35	1	HSV pneumonia (1)
Zinzani-2022	101	3	pneumonitis (1), Epstein-Barr associated lymphoproliferative disorder (1), sepsis (1)

## Discussion

Duvelisib, the world’s first approved oral dual inhibitor of PI3K-δ and PI3K-γ, has been approved for treating patients with R/R CLL/SLL after at least two other treatments in the US and Europe. In Europe and China, duvelisib is indicated for treating adult patients with R/R FL after at least two prior systemic therapies. Recent clinical data on duvelisib suggest a satisfactory efficiency and acceptable safety profile in patients with advanced lymphocyte neoplasms, including R/R CLL/SLL and R/R NHL (23–29). However, the toxicity problems of PI3K inhibitor application cannot be ignored, especially immune-related and infection-related adverse events (47, 54). Unfortunately, at this point the assessments recently reaffirmed by the Panel of experts of the Oncologic Drugs Advisory Committee (ODAC) cannot be omitted, which analyzing the follow-up data of the DUO trial, among others, believes that ‘the risks associated with taking duvelisib by patients with certain blood cancers appear to outweigh the benefits “. So, it is essential to accurately identify the patients benefitting from PI3K inhibitor therapy, and to properly recognize and manage adverse events during treatment. We conducted this meta-analysis and literature review to evaluate the efficacy and safety of duvelisib in different lymphocyte neoplasms, and possible solutions to treatment-relevant toxicity were reviewed. We summarized the results of 11 prospective studies, including 683 patients, 305 patients with CLL/SLL and 378 patients with NHL [187 B-cell iNHL, 39 B-cell aNHL, 152 T-NHL], to assess the safety and efficacy.

This meta-analysis showed that duvelisib could provide satisfactory efficacy in patients with mature B-cell/T-cell Neoplasms. It is worth noting that response rates were particularly noteworthy in patients with RR CLL/SLL and RR iNHL, in whom ORR both were 70%,12-month PFS was 61% and 44%, and 12-month OS was 79% and 77%, respectively. ORR was 49% in RR PTCL patients and 67% in AITL, which are also compelling compared to the poor response rates of other approved single agents. But among the aNHL patients we included, the pooled ORR was only 17%. It’s clear that with the heterogeneity of lymphoid neoplasms, different therapeutic impacts of PI3K-δ and PI3K-γ inhibition could be derived from following aspects.

The first is tumor intracellular impact itself. A key factor is that some lymphocyte neoplasms are highly dependent on maintenance and survival signals from BCR/TCR, chemokine receptors, or co-stimulatory molecules, in which the PI3K-mTOR signaling plays an important role. It’s well known that many mature B-cell malignancies show constitutive PI3K pathway activation because of chronic BCR activation, and are susceptible to kinase inhibitors that disrupt BCR signaling (55–57). In addition, deregulation of the PI3K-AKT pathway has been shown to have a role in the pathogenesis of TCL (46, 58–61). Meanwhile, inhibitors of PI3K-mTOR signaling can effectively treat AITL in preclinical models (46, 58, 62). The second aspect of PI3Kδ inhibition is direct negative impact of the tumor microenvironment on the mitogenic and survival signaling of cells. Inhibition of PI3K-δ induces multiple effects on malignant B-cells, including the arrest of malignant B-cell proliferation and migration mediated by the tumor microenvironment, and inhibition of chemokine secretion derived from tumor cell (44, 56, 63, 64). In some PTCL subtypes, particularly AITL, molecular profiling has elucidated specific microenvironmental signatures associated with poor outcome (65). Of note, the study also shows that AITL presents significant enrichment of B-cell in the microenvironment. These mechanisms can explain why PI3Kδ inhibitors can be used to treat AITL. The third aspect focuses on the fact that inhibition of PI3K-δ, PI3K-γ, or both could activate antitumor immune responses. Indeed, PI3K-δ inhibition enhances antitumor immune response primarily due to a preferential inhibition of immunosuppressive Treg cells in the preclinical model (66–72). while PI3K-γ inhibition reduces the differentiation and migration of key Immunosuppressive cells in the tumor microenvironment, such as M2 tumor-associated macrophages, negatively regulating effector T and natural killer (NK) cells (33, 41, 42).

Despite therapy advances targeting CLL patients (73), CLL/SLL remains incurable (74). Thus, novel and effective agents for R/R CLL/SLL patients are needed. Duvelisib is currently the only FDA-approved PI3K inhibitor for the monotherapy of CLL/SLL. Our meta-analysis revealed that duvelisib could offer reasonable efficacy in patients with RR CLL/SLL without being negatively affected by del17P/TP53 mutation. The pooled ORR of duvelisib was 70%, 12- month PFS and OS were 61% and 79%, respectively. Another approved PI3K-δ inhibitor idelalisib demonstrated only 48% of ORR and 6.9-month median PFS (75). Our data show duvelisib provides improved efficacy over idelalisib. This conclusion is also consistent with previous preclinical CLL models. Dual PI3K-δ, g inhibition has shown stronger activity than blocking either isoform alone (44, 56, 76). Moreover, in a phase 3 randomized study in CLL/SLL, duvelisib demonstrated significantly improved ORR, PFS, and OS compared with ofatumumab (CD20 inhibitor) (24). In addition, duvelisib has also shown high response rates in patients with R/R CLL/SLL who progressed on ofatumumab (77). Many preclinical studies indicate that p53 could inhibit PI3K/AKT/mTOR pathway through multiple targets, mutation or deletion of p53 leading to abnormal activation of these pathways (78–83). Studies show that patients with del(17p) and/or TP53 mutations are more likely to relapse, even when treated with ibrutinib (BTK inhibitor) or venetoclax (BCL2 inhibitor) (84–86). However, duvelisib can prevent CLL/SLL tumor cell proliferation and metabolism by inhibiting abnormally activated PI3K/AKT/mTOR signaling in the context of del(17p) and/or TP53 mutations. Similarly, in our study, ORR,12-month PFS and 24-month PFS, in the subgroup of patients with del(17p) and/or TP53 mutations were similar to those of the whole population, which meant duvelisib could decrease the recurrence due to del(17p) and/or TP53 mutations. Considering the increasing use of ibrutinib and venetoclax in first-line therapy (87–90), duvelisib has been shown to effectively increase the sensitivity of CLL cells to BCL2i and BTKi (91, 92). For patients who are refractory to or intolerable to BTKi or BCL2i, duvelisib is an effective option for RR CLL/SLL with or without del(17p) and/or TP53 mutations.

Although most iNHL patients initially respond to standard chemoimmunotherapy with prolonged remission, eventually, all patients experience disease progression or relapse (93). There are currently several approved options for relapsed or refractory iNHL, but the multiple toxicities of therapies and resistance or transformation to advanced or aggressive lymphomas remain challenges. In our meta-analysis, Response rates of duvelisib were clinically meaningful in patients with RR iNHL across subtypes. In all patients, the pooled ORR was 70%, the 12-month PFS was 44%, and the 12-month OS was 77%. In FL patients, the pooled ORR was 69%. There are already several PI3K inhibitors in clinical trials for RR iNHL patients: copanlisib (intravenous inhibitor of PI3K-α, -β), idelalisib (oral inhibitor of PI3K-δ), umbralisib (oral inhibitor of PI3K-δ, CK-e). ORR in these trials ranged from 47% to 59% in all patients and 45% to 59% in FL patients (94–96). Despite heterogeneity in cross-trial patient selection and prior treatments, our data reveal that duvelisib had higher efficacy than other PI3Ki treatments. Repeating chemotherapy, even combined with different CD20 antibodies like Obinutuzumab, caused cumulative toxicities and decreased efficacy. In our study, almost all RR iNHL patients had been previously treated with rituximab (100%, 94%, 100%, and 100%, reported in 4 studies, respectively) or alkylating agent (98%, 81% reported in 2 studies, respectively). Given the increasing use of rituximab and alkylating agents for the untreated or RR iNHL, duvelisib monotherapy may provide an option for R/R patients. Additionally, PI3Kδ inhibition restores the sensitiveness of FL cells on the anti-apoptotic protein BCL-2 (63), showing a rationale potent for combined PI3Kδ and BCL-2 inhibition.

In the aggressive B-NHL, the pooled ORR was 68% in MCL and 17% in other aNHL (Mainly DLBCL), respectively, while in copanlisib, the ORR of the aggressive cohort ranged from 7% in DLBCL patients to 64% in MCL patients (97). The responses to ibrutinib have been reported at 37% in ABC DLBCL patients and 5% in GCB DLBCL patients. Although no data on DLBCL subtypes is available here, considering that ABC DLBCL often selectively acquires mutations targeting B-cell receptors (BCRs) that promote chronically active BCR signaling (98), duvelisib may be a rationale candidate for ABC DLBCL. Data from a phase 1 study have demonstrated that the combination of duvelisib with standard therapies, bendamustine, and rituximab, is well tolerated and presents a novel therapeutic option in B-NHL, including DLBCL and MCL (99).

Peripheral T cell lymphomas (PTCLs) are highly heterogenous diseases with a poor prognosis. Immunotherapy and novel chemotherapy protocols are active in B cell lymphomas, unluckily with a high failure rate and frequent relapses in T-NHL. Indeed, new treatments for peripheral T cell lymphomas (PTCLs) are developing, but patients with PTCLs still have poor survival. In our analysis, the pooled ORR was 49% in patients with RR PTCL, which is also satisfactory, for the response rates of other approved single agents for RR PTCL, like romidepsin (HDACi), belinostat (HDACi), and pralatrexate (antifolate), range from 25% to 29% (20, 100, 101). And, for another PI3K inhibitor copanlisib, the ORR of PTCL was only 21% (97). Additionally, a phase I study of duvelisib combined with romidepsin, has shown a better ORR(58%) than previous therapy using romidepsin alone (102). In the subgroup analysis of the PTCL subtype, we found that AITL patients appeared to have higher pooled ORR than other PTCL patients (67% vs. 42%, p=0.01). The similar results could be observed in phase I study of duvelisib combined with romidepsin, AITL has also shown a better ORR than other PTCL (68% vs. 53%). Meanwhile, we also noticed a high pooled PDR of 47%, also observed in copanlisib (36%) for all PTCL. However, in one of our studies included patients with AITL had a lower PDR (0 of 3) compared to other PTCLs (6 of 13) (33). These results suggested the existence of heterogeneity between diseases. Long-term outcomes of retrospective series, such as the International T-cell lymphoma project (ITCP), displayed that the 5-year failure-free survival (FFS) for the AITL patients receiving CHOP was only 18%. Therefore, more novel therapies should be explored for T-NHL. Duvelisib would be a good option for patients with RR PTCL, especially AITL. Given the phenomenon observed in our study, clinical trials with larger sample size were needed to characterize more details of differential efficacy of duvelisib in PTCL subtypes.

PI3K-δ, g dual inhibition significantly changes the cellular composition of the microenvironment by reducing Treg cell numbers and activating CD4+ and CD8+ cells, which clonally expand and display enhanced cytotoxic and cytolytic properties. Despite enhancing anti-tumor immunity, activated T cells invariably cause immune dysregulation in normal tissues. The safety profile in the individual disease subgroup was found to be generally consistent with the subjects. Nearly all patients in both subgroups experienced an AE. AEs were generally low grade and manageable, probably leading to dose reductions/interruptions, among which 33% of patients discontinued treatment, similar to observations with other PI3K-δ inhibitors (95, 103). Immune-related toxicities are common, leading cause of treatment discontinuation, including transaminase elevations, diarrhea or colitis, pneumonitis, and rash. In our meta-analysis, diarrhea and transaminase elevations were the most frequent non-hematologic AEs (47% and 39%, respectively) and the most common grade≥3 non-hematologic events (12% and 16%, respectively) in all diseases, which could be managed by adjusting dose, discontinuing and recovering treatment when monitoring hepatic function. Only 1 patient was reported to die of treatment-induced pancolitis. Similar results have been observed in other PI3K inhibitors. The incidence of diarrhea in any grade and grade ≥3 was 43% and 13% in idelalisib, and 34% and 5% in copanlisib, respectively. For transaminase elevations, the incidence in any grades and grade ≥3 were 47% and 13% in idelalisib, 28% and 2% in copanlisib, respectively (94–96). The subanalysis showed the incidence of grade≥3 spoke of potential differences among subgroup patients. This difference may be due to toxic modulation by disease-specific factors, or different prior treatments, or differences in immune cell populations. Consistent with this difference, elevated transaminases are a major factor for treatment discontinuation compared to diarrhea in NHL, whereas in CLL/SLL, the opposite could be witnessed.

Pneumonitis and rash, which are also common in the application of idelalisib and copanlisib, are the other leading cause of treatment discontinuation. Grade≥3 pneumonitis occurred in 6% of patients, including 1 fatal thought to be duvelisib relevant. Some patients experienced skin toxicity at any grade (rash 18%, rash maculo-papular 19%), and greater than grade 3 are uncommon (rash 2%, rash maculo-papular 5%) but still cause 2 casualties, which are duvelisib relevant. Duvelisib has a satisfactory hematological safety profile. The most frequent hematologic AEs were neutropenia (38%) and anemia (21%). Notably, neutropenia was the most frequent severe AEs (25%). The incidences of neutropenia and anemia in other PI3K inhibitors were similar to our results (94, 95). These events seldom require treatment modifications due to their reversibility, and rarely result in treatment discontinuations (neutropenia in 1 patient).

Grade≥3 serious infections occurred in 19% of patients treated with duvelisib. Infectious complications are major causes of mortality in duvelisib treatment, as a result of the humoral immunodepression inherent to the disease and therapy-induced immunosuppression (104, 105). A total of 17 treatment-related deaths in 577 patients (pooled rate of 3%) were reported in our included studies, 12 (pooled rate of 2%) were infection-related. These were similar in copanlisib (3/142,1 infection-related) and idelalisib (8/125, 4 infection-related). Severe pneumonia occur in 10% of patients, and few are fatal, 5 of which were assessed as related to duvelisib: staphylococcal pneumonia (n=2), pneumocystis jirovecii pneumonia (PJP), respiratory syncytial viral pneumonia, and HSV pneumonia (n=1 each). If pneumonias are suspected, appropriate and extensive evaluations should be performed for infectious etiologies of pneumonias. In clinical trials, many patients have been treated with antibiotics and corticosteroids and most recovered. Prophylaxis for PJP infections is required to mitigate the risk of these opportunistic infections, which have been reported in other B-cell receptor inhibitors (106–109). And antiviral prophylaxis also should be implemented at the consideration of the clinicians.

Generally, duvelisib is effective in treating mature lymphocyte neoplasms. Still there are significant patients requiring dose adjustments, and up to 33% of patients discontinue the treatment because they could not tolerate the AE (41% in CLL/SLL, 27% in B-NHL and T-NHL). A reasonably designed intermittent dosing regimen may help reduce the incidence of AEs without compromising efficacy. The TEMPO study (NCT04038359) evaluates the effects of duvelisib prespecified 2-week dose holidays on responses and safety/tolerability in patients with iNHL (110). Similarly, in mouse models, a modified treatment with intermittent dosing of PI3Kδi led to a decrease in tumor growth without inducing pathogenic T cells in colonic tissue, indicating that alternative dosing regimens might limit the toxicity of colits (111). What’s more, extended survival is observed in patients who had treatment interruptions with the PI3Kδ inhibitor idelalisib in FL and CLL, indicating that discontinuous PI3Kδ therapy may also achieve clinical benefit (112).

Due to the differences in study design and patient enrollment, it is difficult to compare the risk of immunotoxicity directly. between different PI3K-δ inhibitors. However, compared with idelalisib, which only inhibits PI3K-δ, duvelisib may reduce autoimmune complications through the simultaneous inhibition of PI3K-γ based on preclinical data, preventing leukocyte recruitment and reducing dextran sulfate sodium-induced colitis in mice (53). Pharmacological blockade of PI3K-γ suppresses joint inflammation in mouse models of rheumatoid arthritis and eases inflammation in a model of colitis-associated cancer (113, 114). Further studies to elucidate the effect of PI3K-γ on the immune system during duvelisib treatment are of great interest. Another intravenous PI3K-δ/α inhibitor copanlisib, has a specific AE profile, including hypertension and hyperglycemic effects mediated by PI3K-α isoform inhibition, limiting its application in elderly patients with a high prevalence of these comorbidities. And hospital visit for infusional therapies represents an essential concern for some people, who are likely to benefit significantly from oral drug treatment.

There are still some limitations in our study. Firstly, most of the studies involved were single-armed studies without double-blinded randomized controlled trials, which may lead the potential performance bias. In addition, a small number of patients received different drug doses, and some AEs may be dose-dependent. Finally, although the diseases were all relapsed or refractory, the degree of lymphoma and leukemia was dispersive, which might have caused bias in the final analysis and made comparisons with other studies difficult. No results of survival benefit were defined in the study due to the varied length of follow-up time and survival data in some studies shown incompletely.

In conclusion, our analysis shows that duvelisib is an effective monotherapy for RR mature lymphocyte neoplasms. Duvelisib could offer favorable efficacy in patients with RR CLL/SLL and is not negatively affected by del17P/TP53 mutation. Besides, duvelisib has better efficacy than other approved PI3K inhibitors in iNHL treatment, including FL. And duvelisib monotherapy shows unexpectedly good efficacy in PTCL, especially in AITL. However, the efficacy of duvelisib in aNHL was limited. Although fatal and severe toxicity occasionally exists, risk and severity in duvelisib treatment have the potential to be mitigated through identification and management properly. Based on the limits of the single-arm studies, more randomized controlled studies are needed to explore the efficacy and safety of duvelisib with or without combination with other drugs for patients with RR lymphoma and leukemia.

## Data availability statement

The original contributions presented in the study are included in the article/**Supplementary Material**. Further inquiries can be directed to the corresponding author.

## Author contributions

ZW and HZ have contributed equally to this work and share first authorship. ZW and HZ participated in the study design. ZW and HZ acquired, analyzed and interpreted the data. ZW wrote the manuscript. HZ and TN revised the manuscript. JX proofread the language. JW provided methodological advice. All authors contributed to the manuscript and approved the submitted version.
