# Supplementary material for: Safety and efficacy of dual PI3K-δ, γ inhibitor, duvelisib in patients with relapsed or refractory lymphoid neoplasms: A systematic review and meta-analysis of prospective clinical trials
Source: Front Immunol. 2023 Jan 4;13:1070660. doi: 10.3389/fimmu.2022.1070660 (PMC9845779; doi:10.3389/fimmu.2022.1070660)

Supplementary Material

Supplementary Table 1. The MINORS scores of 10 single arm studies

| **Study** | **1** | **2** | **3** | **4** | **5** | **6** | **7** | **8** | **Total** |
| --- | --- | --- | --- | --- | --- | --- | --- | --- | --- |
| O'Brien-2018 | 2 | 2 | 2 | 2 | 0 | 2 | 2 | 0 | 12 |
| Flinn-2018 | 2 | 2 | 2 | 2 | 0 | 2 | 2 | 0 | 12 |
| Davids-2020 | 2 | 2 | 2 | 2 | 1 | 2 | 2 | 0 | 13 |
| Flinn-2019 | 2 | 2 | 2 | 2 | 1 | 2 | 2 | 0 | 13 |
| Zheng-2021 | 2 | _ | 1 | 2 | 1 | 2 | 2 | _ | 10 |
| Izutsu-2020 | 2 | 2 | 2 | 2 | 0 | 2 | 2 | 0 | 12 |
| Flinn-2018 | 2 | 2 | 2 | 2 | 0 | 2 | 2 | 0 | 12 |
| Horwitz-2018 | 2 | 2 | 2 | 2 | 0 | 2 | 2 | 0 | 12 |
| Horwitz-2019 | 2 | _ | 1 | 2 | _ | 2 | _ | _ | 7 |
| Zinzani-2022 | 2 | _ | 1 | 2 | _ | 2 | _ | _ | 7 |


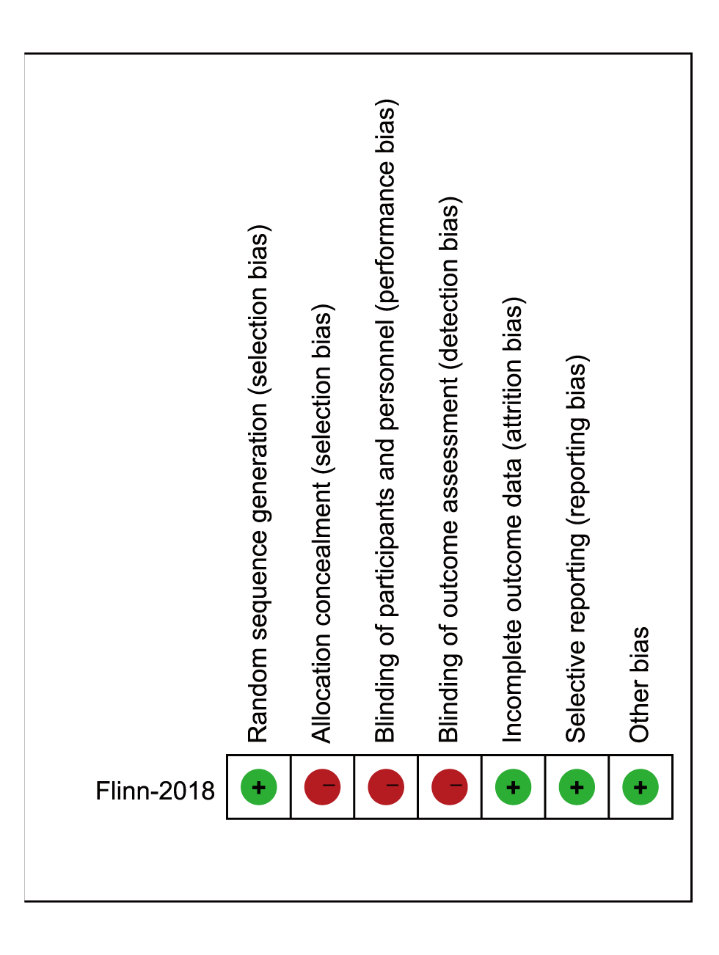
**Supplementary Figure 1.** The bias of the 1 included RCT study assessed by the Cochrane Collaboration Risk of Bias Tool

**Supplementary Figure 2.** The forest plot of pooled ORR (A), SDR (B) and PDR (C) of T−cell non−Hodgkin lymphoma (T-NHL).


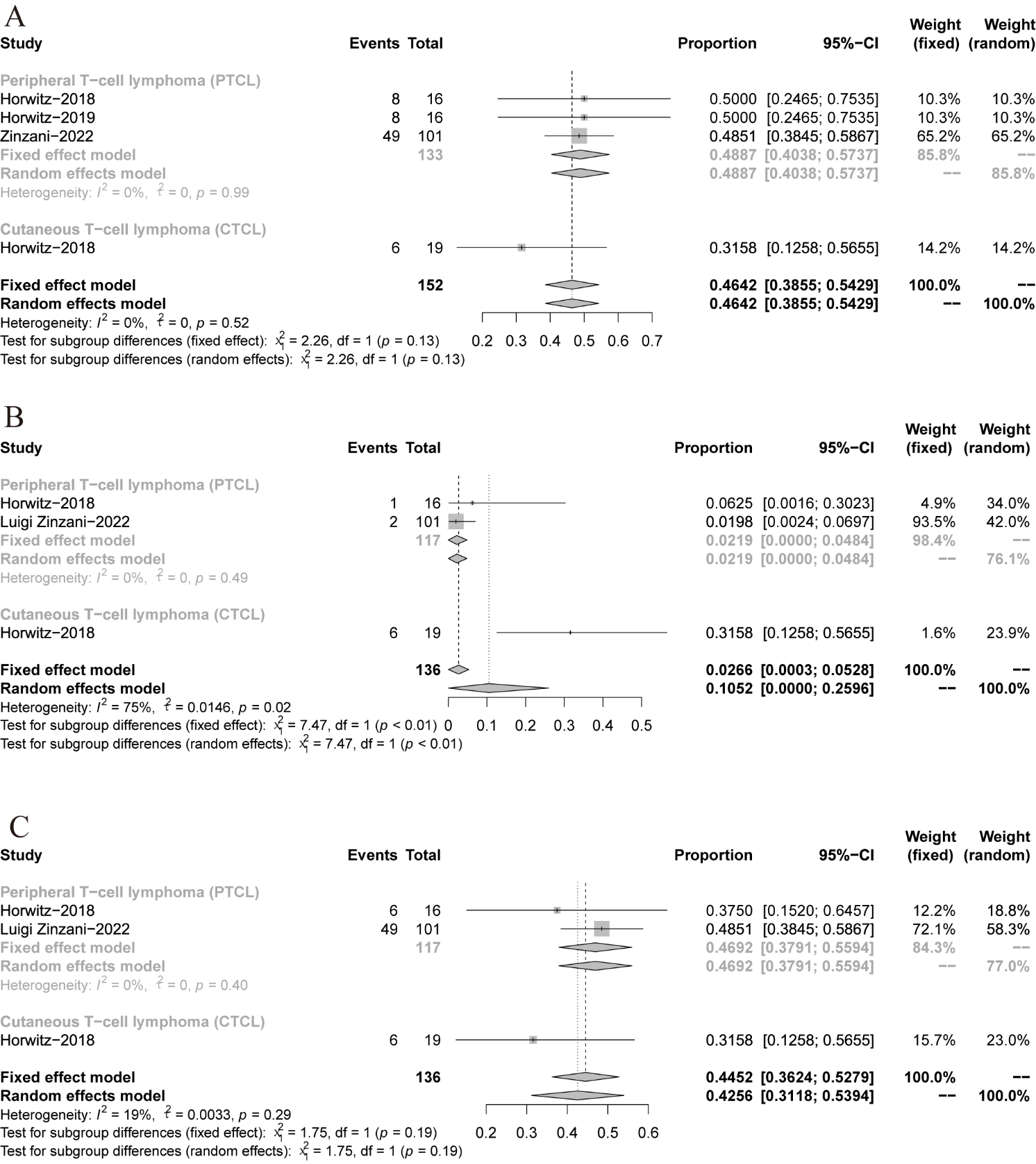


**Supplementary Figure 3** The forest plot of pooled SDR (A) and PDR (B) of Chronic lymphocytic leukemia / small lymphocytic lymphoma (CLL/SLL), Indolent non−Hodgkin lymphoma (iNHL), Aggressive non−Hodgkin lymphoma (aNHL) and T−cell non−Hodgkin lymphoma (T-NHL).

**
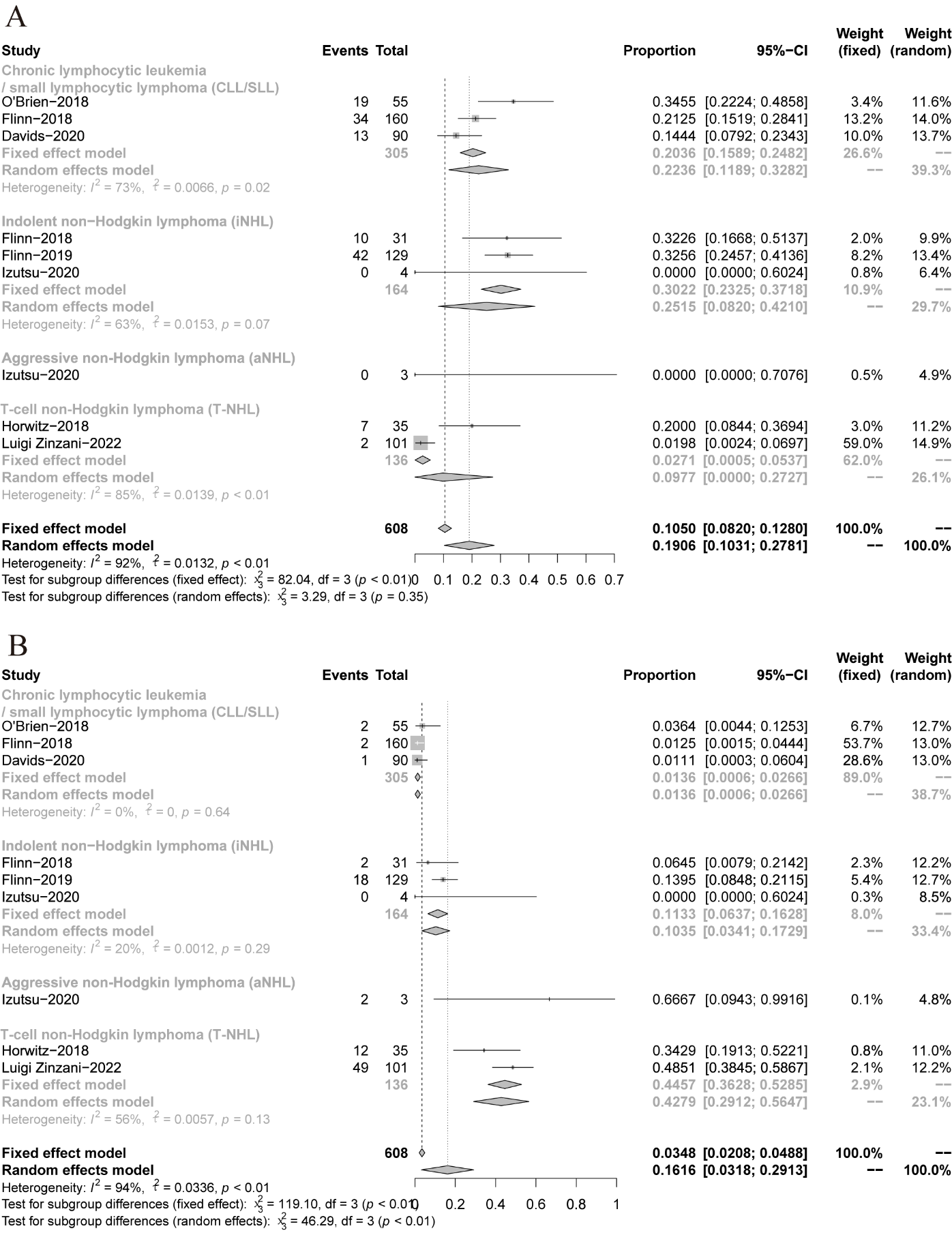
.**

**Supplementary Figure 4** The forest plot of pooled 24-month PFS rate of CLL/SLL (A) and CLL/SLL with TP53 mutation/17p-deletion (B).

**
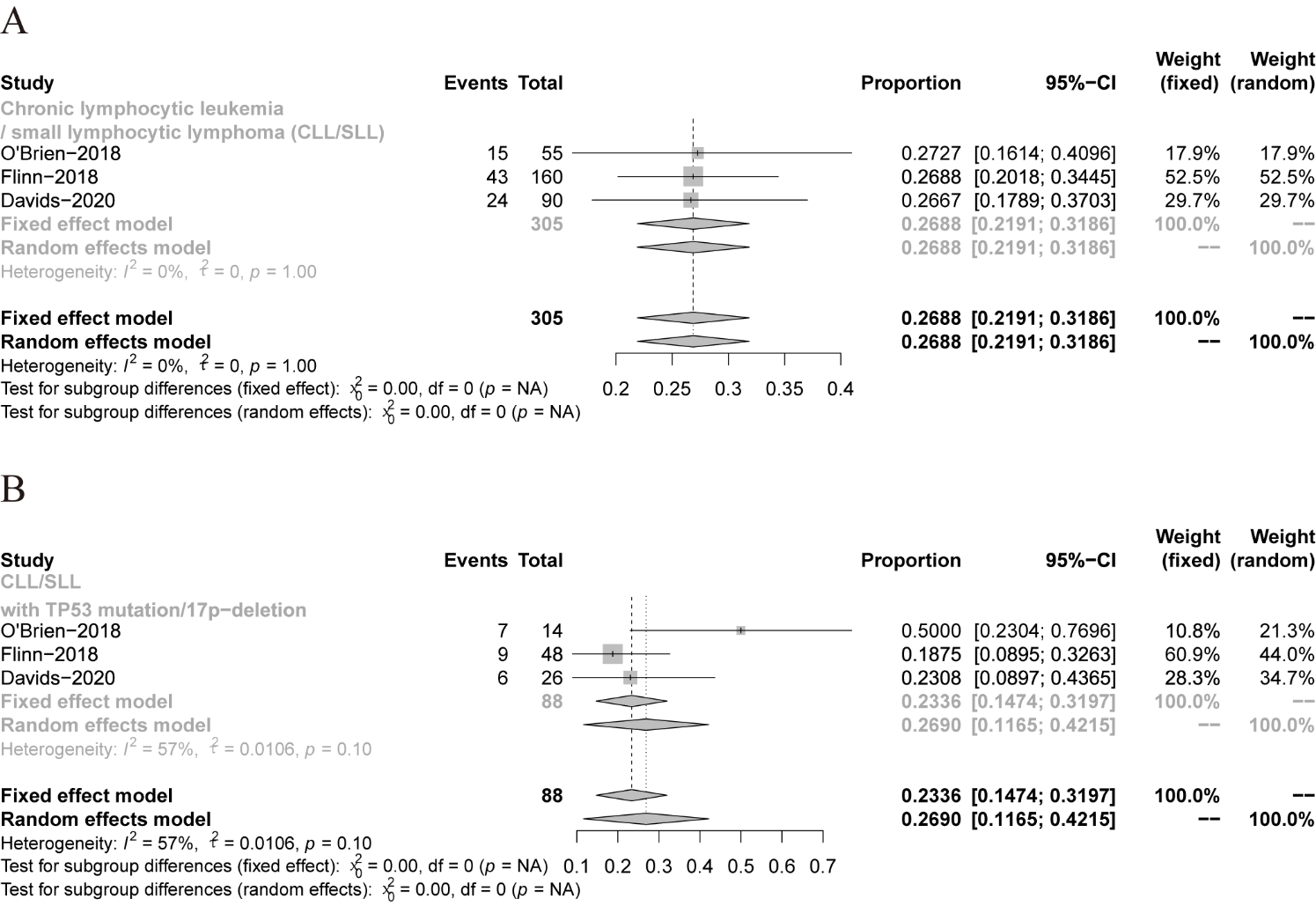
**

**Supplementary Figure 5** The AEs most frequently leading treatment discontinuation. Including transaminase increase (A), colits (B) and diarrhea (C).


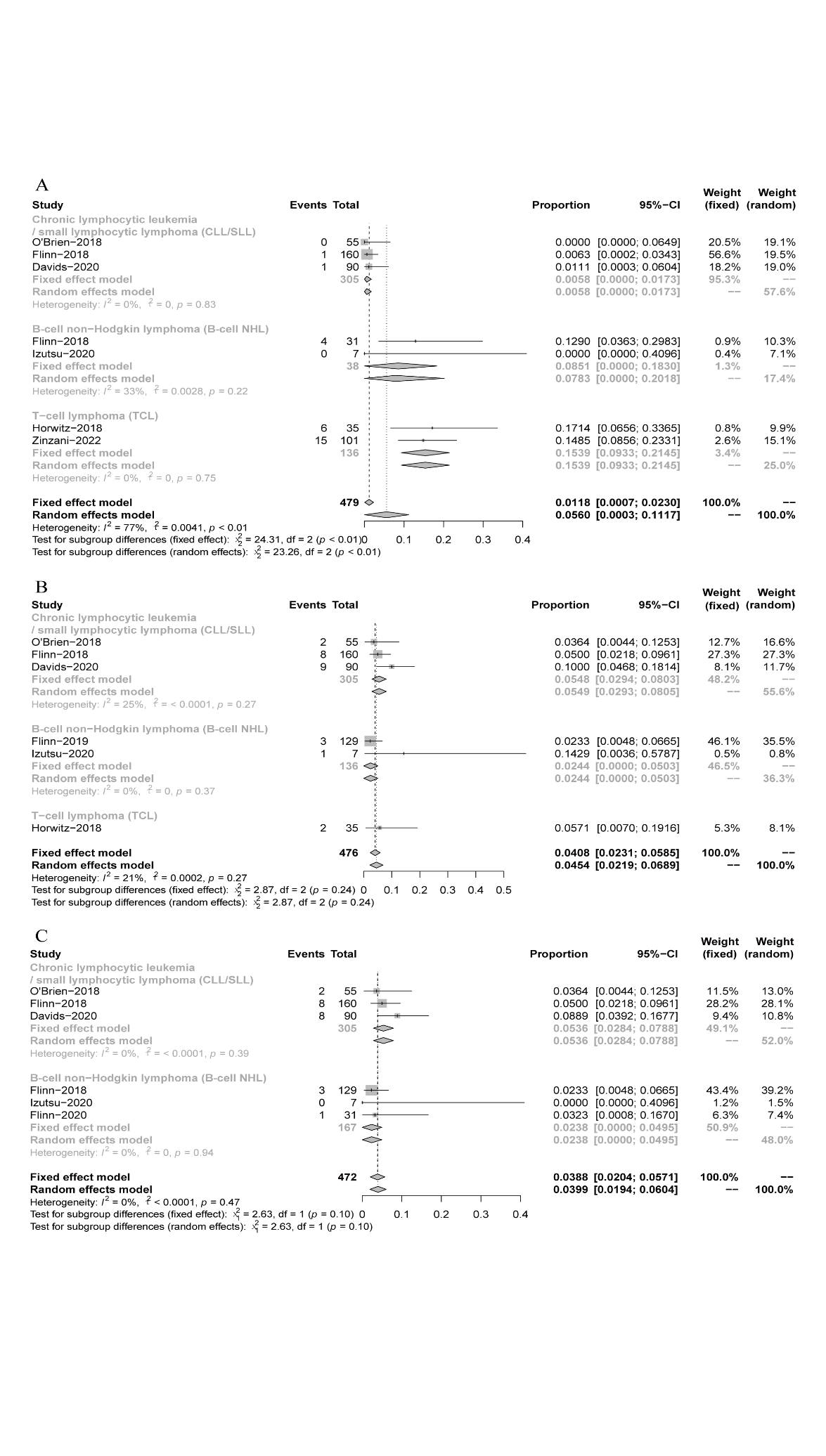

Supplement: Supplementary file 1 [file DataSheet_1.docx]
